# Supplying the trip to antibody production—nutrients, signaling, and the programming of cellular metabolism in the mature B lineage

**DOI:** 10.1038/s41423-021-00782-w

**Published:** 2021-11-15

**Authors:** Mark R. Boothby, Shawna K. Brookens, Ariel L. Raybuck, Sung Hoon Cho

**Affiliations:** 1 Department of Pathology, Microbiology & Immunology, Molecular Pathogenesis Division, Vanderbilt University Medical Center, Nashville, TN 37232 USA; 2 Department of Medicine, Rheumatology & Immunology Division, Vanderbilt University Medical Center, Nashville, TN 37232 USA; 3 Cancer Biology Program, Vanderbilt University, Nashville, TN 37232 USA; 4 Vanderbilt Institute of Infection, Inflammation, and Immunology, Nashville, TN 37232 USA

**Keywords:** B lymphocyte, Plasma cell, Intermediary metabolism, Glucose, Glutamine, Fatty acid, Signal transduction, Germinal centres, Somatic hypermutation

## Abstract

The COVID pandemic has refreshed and expanded recognition of the vital role that sustained antibody (Ab) secretion plays in our immune defenses against microbes and of the importance of vaccines that elicit Ab protection against infection. With this backdrop, it is especially timely to review aspects of the molecular programming that govern how the cells that secrete Abs arise, persist, and meet the challenge of secreting vast amounts of these glycoproteins. Whereas plasmablasts and plasma cells (PCs) are the primary sources of secreted Abs, the process leading to the existence of these cell types starts with naive B lymphocytes that proliferate and differentiate toward several potential fates. At each step, cells reside in specific microenvironments in which they not only receive signals from cytokines and other cell surface receptors but also draw on the interstitium for nutrients. Nutrients in turn influence flux through intermediary metabolism and sensor enzymes that regulate gene transcription, translation, and metabolism. This review will focus on nutrient supply and how sensor mechanisms influence distinct cellular stages that lead to PCs and their adaptations as factories dedicated to Ab secretion. Salient findings of this group and others, sometimes exhibiting differences, will be summarized with regard to the journey to a distinctive metabolic program in PCs.

## Preface

Along with resistance to the effects of microbes that undermine reproductive fitness, nutrient supply is a second major limiting factor in Darwinian selection. These two factors related to fitness selection are linked in part through sensing of cellular nutrients or whole-body metabolism. Such mechanisms act within cells at each step after emergence of the mature B lineages, leading to the survival benefits that accrue from having appropriate concentrations, locations, and specificities of antibodies. Given the importance of these mechanisms of protection and their centrality to the efficacy of vaccines, the amount of literature on this interplay in B cells or plasma cells is remarkably small compared to the amount of literature on T cell helpers and other types of T cells. Nonetheless, important insights from B cell ontogeny will be omitted here, as will autoimmunity. Several excellent general reviews are sufficiently recent to provide overviews of metabolism in B cells [1–5] and plasma cells [6, 7]. This article will strive instead to provide an account of the stages on the road from naive B cells to intermediates to Ab-secreting plasma cells, adding consideration of work from the past few years in these areas and topics less covered in standard reviews to the existing foundation. As an understudied area at the frontier, the topic covered in the current work involves papers that potentially contradict one another, and consideration will be given to potential models that could account for the differences. In addition to presenting the content of disparate publications, efforts will be made to highlight open questions and moot possibilities that verge on the speculative. These will generally be marked by different conventional English tenses to distinguish generally accepted or amply replicated information (present tense), reported findings (past tense), and possibilities (a conditional voice or verb tense, without use of an active past tense). A cognitive bias in the background is the view that evolution and Darwinian fitness are likely to favor diversity—even within one individual and certainly among immunogens and individuals. This point underscores the importance of eliminating a cultural tendency to state a body of results as being universally true despite strong evidence of variety in each class of cells and locale.

## Introduction and overview

Pre- and postnatal ontogeny yield three classes of B cells that can progress to antibody secretion—the B1 lineage and 2 B2 lineages, follicular (FO), and marginal zone (MZ) B cells [8, 9]. However, these classes exhibit differences in their functions and molecular programs [9, 10]. B1 B cells, subdivided into B1a and B1b subsets, are thought to be the predominant sources of circulating immunoglobulins (Igs) termed natural antibodies, which arise without overt immune challenge [9, 11, 12]. For simplicity, this review will treat B1a and B1b cells collectively as “B1 B cells” despite differences between the two types. In contrast to the B2 subset, a fraction of B1 cells appears able to reprogram splicing to generate secreted natural antibodies without expression of the transcription factor Blimp1 [10]. Moreover, B1 cells are major sources of T-independent (T-I) antibodies, which rely less on interaction with or help from CD4^+^ T cells for secretion than other antibodies [11–14]. B1 cells are widely distributed, including in lymphoid organs, but the peritoneal cavity is a major site of residence [8, 15]. Peritoneal B1 cells serve in part as precursors to a dense population of IgA-secreting plasma cells in the intestines [16–20]. Only the lining of this potential space is vascularized so that fluid within permeates from plasma as a transudate unless infection is present [21, 22]. Since splenic B1 cells can participate in a rapid (i.e., within 3 days) wave of Ab production—for instance, after exposure of the host to particulate antigens (Ags)—they have been viewed as “endowed with a ‘natural memory’” that in combination with MZ B cells “provide(s) a bridge between the very early innate and the later-appearing adaptive immune response” [11, 13]. A broad property of more robust and higher-affinity Ab production following a recall challenge may be less evident for T-I type II responses—which by inference may be B1-derived—than is characteristic of the follicle-derived B2-based response [23, 24]. Nonetheless, there is evidence of adaptive characteristics such as somatic hypermutation (SHM) and the formation of memory-phenotype cells that can respond to later stimulation with the same antigen [14, 23–27], although the evidence is quantitatively and qualitatively less robust than that for B2-lineage cells.

**Fig. 1:**
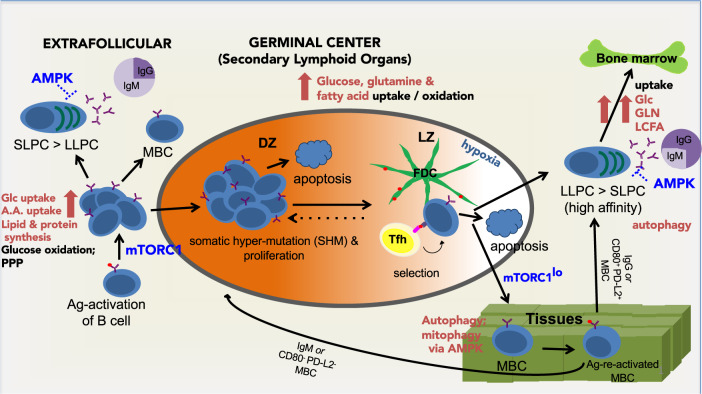
Simplified schematic of B cell routes to antibody secretion and humoral memory. Shown is a representation of progress along the B lineage along with limited highlights of metabolic regulators and changes in programming of intermediary metabolism in stages past the quiescent naive B cell stage (lower left) after antigen activation. The steps have been discussed in detail throughout this review, and more background on the signals and gene expression programs has been provided in earlier reviews [1–3, 56, 57]. For simplicity, issues unique to B1 and marginal zone B cells are omitted here. Successful BCR engagement and costimulation along with extrafollicular T cells help lead to increased cell mass and rounds of proliferative expansion that require large mTORC1-mediated increases in precursor uptake, macromolecule synthesis, energy generation, and maintenance of redox balance (middle left side). High-affinity BCR facilitates extrafollicular plasma cell generation (short- and long-lived plasma cells, i.e., SLPCs and LLPCs), with AMPK then restraining rates of protein synthesis (upper left), but memory B cells (MBCs) can also arise. Among the activated B cells, some with cognate help may move into the germinal center (GC) reaction that occurs in secondary follicles (middle of diagram). After a round of T cell help, proliferation, AID-induced mutations, i.e., somatic hypermutation (SHM), and p53-mediated apoptosis from genotoxic stress occur in the dark zone (DZ). Surviving progeny (~50%) move to the light zone (LZ), in which their BCRs can compete for capture of antigens from stromal cells (follicular dendritic cells (FDCs)), which can trigger apoptosis in the absence of help but allows internalization, epitope presentation on MHC-II, and enlistment of T cells. Apart from death and continuation in the GC, these B cells can assume a quiescent state that probably involves some degree of differentiation as MBCs (which can be subdivided according to IgM or CD80 and PDL2), some of which circulate to tissues. Alternatively, the cells can acquire a plasmablast/plasma cell fate in which IgG^+^ PCs supported by stromal niches can persist for months to years as LLPCs in the bone marrow. As discussed in the text, MBC persistence is promoted by both AMPK and canonical autophagy, whereas LLPC persistence appears to be autophagy-dependent but AMPK-independent

MZ B cells in mice that are positioned initially near the marginal sinus, where stimulation by antigens displayed on particles is particularly favored, provide a second source of plasma cells. Similar to B1 cells, the MZ B population can yield rapid, “innate-like” plasmablasts and antibody responses [11, 13]. Alternatively, MZ B cells can migrate to secondary lymphoid follicles, i.e., germinal centers (GCs), after activation [28]. Apart from MZ B cells, the majority of splenic and lymph node B cells initially have an FO phenotype. One canonical pathway for FO B cells involves activation, recruitment of help from CD4^+^ T cells, proliferation, and relatively rapid differentiation into plasmablasts and then plasma cells in an extrafollicular location [29–31] (reviewed in [32]). An alternative fate, also dependent on T cell help, involves migration to sites defined by specialized stromal cells that secrete suitable chemoattractant and positioning signals and establish GCs (reviewed in [33–35]). The affinity of the B cell antigen receptor (BCR) for the epitope that initiates B cell activation is a major factor that tilts the balance toward either extrafollicular plasma cell generation or earlier Ab secretion or GC B cell fate acquisition [30, 36, 37] (Fig. 1). In T-independent responses, activation requires higher affinity, while the T-dependent process is regulated by high-to-intermediate affinity of BCR binding to the antigen [36, 37]. Although beyond the scope of this review, it is crucial to note that the processes are also regulated by stimuli triggered in B cells through interaction with follicular helper T (Tfh) cells, such as CD40 engagement by CD40L (CD154) on Tfh cells and secretion of cytokines such as IL-4 and IL-21 (reviewed in [38–40]).

Both the T-independent and extrafollicular roads to Ab-secreting cells yield protective responses, immune memory, and plasma cells [41–43], but GC reactions and the fates of their B cells offer advantages for vaccines and likely in evolutionary selection. With extensive proliferation and vastly elevated expression of the enzyme AID, the DNA sequences encoding each BCR of a GC-immigrant B cell diversify substantially, leading to changes in affinity and even specificity [44–47] (reviewed in [48–51]). This process of SHM can shift the universe of circulating Abs toward greater affinities when coupled with selection for GC B cells with high-affinity BCR to capture antigen, present it on their surface MHC-II after internalization and processing, and thereby restimulate Tfh cells to receive new help (e.g., CD40L and IL-21) in an iterative process (reviewed in [33–35, 38–40]). The progeny of B cells that enter GC reactions follow four main fate pathways: (1) death from genomic damage or from a failure to be selected [52]; (2) resumption of quiescence with the characteristics of a memory B cell subset ([53–56]; reviewed in [57]); (3) retention and persistence in the GC [55]; and (4) differentiation to plasmablasts and later plasma cells (e.g., [58], reviewed in [57]). In this last case, transcription factors that define B cell identity (Pax5) and the GC B cell state (BCL6) are replaced by Blimp1 and heightened levels of IRF4 (reviewed in [59, 60]). Among other actions, Blimp1 enhances Ig gene transcription rates by over ten-fold and drives the expression of ELL2, an RNA processing factor that supports reorganization of splice choice in the Ig heavy chain gene transcript to convert from surface BCR expression to antibody secretion [61–63]. Periods of migration and issues of positioning or localization are intrinsic to all the steps involved in antibody responses summarized above (e.g., [35, 59, 63–66]. The different sites at which plasmablasts and plasma cells reside are an example of how considerations of nutrient supply to and metabolic programming of mature B lineage cells and the Ab-secreting populations at their final destinations include the potential for diversification of these factors at distinct sites. In the sections that follow, the issues of nutrients and metabolic programming will be summarized with regard to the conversion from resting to initial lymphoblasts (section “Blasting off from the resting state”), decisions about fate choice after activation (section “Decisions, decisions—B cells after activation”), and the emergence and persistence of plasma cells (section “The end of the affair—building and fueling the antibody factories”). Because signal transduction regulates metabolism and because metabolites modulate signaling, information on a few selected aspects of signaling will be included in these sections.

## Blasting off from the resting state

**Fig. 2:**
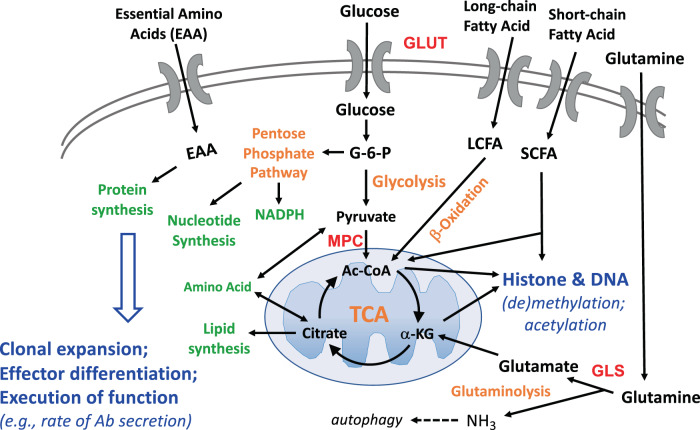
Nutrient uptake and usage by pathways of intermediary metabolism linked to downstream processes. Simplified schema of items discussed in more detail in the body of the text. The extracellular milieux in which B lineage cells reside and through which they pass, in the upper portion of the diagram, may differ in concentrations of key constituents that include glucose, glutamine, essential amino acids (EAAs, i.e., those that cannot be synthesized in the B cells), and fatty acids (both short- and long-chain, i.e., SCFAs and LCFAs). The multiplicity of different transporters used for import (and in some cases export) of these nutrients is omitted from the picture, but as noted in the text, glucose may pass through at least three different molecules whose ratios may be different depending on the B lineage cell type, while glutamine has over four different routes. Several amino acids in addition to glutamine can be fed into mitochondria and the Krebs (TCA) cycle. The branch-point between glycolysis and the pentose phosphate shunt pathway at glucose-6-phosphate (G-6-P) is shown along with the mitochondrial pyruvate channel (MPC) as one route for pyruvate entry and conversion to acetyl-coenzyme A (Ac-CoA), but additional diversions of metabolites prior to ending glycolysis as pyruvate may be possible and are not shown. A suitably balanced combination of protein, nucleotide, and (phospho)lipid synthesis is required for clonal expansion, effector differentiation, and the execution of functions such as secretion of glycosylated antibody molecules, all of which also require energy (ATP)

Plasma cell development and Ab production requires multiple divisions after initial activation of a B cell that is quiescent in G0, with maintenance of osmotic equilibrium and membrane potential as well as quality control that involves protein turnover. An early phase involves a combined challenge of rapidly generating new protein and lipid biomass along with ribonucleotides and deoxyribonucleotides for new ribosomes and doubling nuclear DNA to license each division after B cell activation. In parallel, these processes—increased rates of protein and polymeric nucleic acid synthesis—add to the demand for energy and rates of ATP generation. Moreover, flux through pathways that feed these processes tends to require interconversion between oxidized and reduced forms of NAD(P)^+^ and NAD(P)H in each subcellular compartment (e.g., the cytosol vs. mitochondria). Accordingly, a challenge at each stage is to tune flux in these pathways so that the use of favored building blocks such as glucose, amino acids, and fatty acids (Fig. 2) achieves growth needs while maintaining tolerable redox ratios and pools of ATP and GTP. Current limitations in the ability to quantify these processes in vivo make in vitro analyses essential but potentially misleading.

### Signals and substrates for biosynthesis

Naive B cells maintain a survival program that requires signals derived both from their BCRs and stimulation of the receptor for B cell activating factor (BAFF-R) (reviewed in [1–3]). After mitogenic stimulation through their BCRs, coreceptors, and/or TLRs (reviewed in [2, 3]), they greatly increase glucose uptake and generate energy both by glycolysis and by glucose oxidation (i.e., by feeding the Krebs cycle using the pyruvate produced by glycolysis [67–71]). Using tritiated palmitate, FAO was also detected with resting B cells in vitro; unlike glucose use, this process appeared IL-4-independent [70]. The initiation of signaling to maintain metabolism-linked fitness is an expected function of the BCR-linked protein tyrosine kinase Syk [72, 73] and has been reviewed previously [74]. In addition to this pathway, newer work provides evidence of a novel Syk-independent means by which the BCR complex is sensed in the endoplasmic reticulum (ER) to then signal to mitochondria [75]. Nonetheless, judging from “metabolic flux” (i.e., Seahorse) assays, the quiescent naive B cell has a relatively low respiration rate and little lactate excretion ([76], and data in [77–79]). Interestingly, a very modest but statistically significant increase in respiration was observed in Seahorse measurements 90 min after BCR crosslinking by anti-IgM or TLR9 stimulation, accompanied by prevention of the decline in an already-low ECAR [76]. Maintaining proper rates of glycolysis and oxidative glucose metabolism is likely essential for normal naive B cell survival, as elimination of the von Hippel–Lindau protein to stabilize the hypoxia-inducible transcription factor HIF-1 accelerated apoptotic death and reduced the number of mature B cells [80, 81]. This loss appeared to depend on an imbalance of metabolic pathways that led to excessive levels of the pro-death BH3-only protein Bim [81] and activation of caspase 8 [80]. Conversely, some evidence indicated that an initial increase in respiration and presumably mitochondrial function driven by BCR crosslinking needed a costimulatory signal from CD40 or TLR9 to be maintained on the first day [76].

One could speculate that rates of protein turnover (which requires energy for new protein synthesis) are low and that autophagy could reduce the need for synthesis of new phospholipid mass. A reported requirement for the product of the essential autophagy gene *Atg5* for normal B1a and B2 cell numbers would be consistent with this model [82]. However, disparate findings exist: inactivation of *Atg5* or *Atg7*, another crucial gene for the process, with different Cre drivers was reported to decrease B1a but not conventional B2 B cell numbers ([83, 84]; reviewed in [85, 86]). Collectively, the papers suggest that the development or maintenance of B1 B cells is more dependent on autophagy than that of the B2 lineage.

Activated B cells can import exogenous nucleic acid precursors, as inferred from in vivo incorporation of compounds such as bromodeoxyuridine and relatives such as EdU (e.g., [51]). In addition, uptake of glucose increases substantially—measured either directly and rigorously [67–71] or with a bulky fluorogenic analog, 2-NBDG [77, 87]. This latter facilitates estimates based on intravital uptake and cell labeling, but recent evidence shows that uptake of 2-NBDG can differ substantially from that of glucose [88, 89]. Two crucial observations were reported by a pioneer in metabolism studies with primary B cells [67]. First, B cell activation-induced increases in glucose uptake and utilization led to substantial use of glucose by the oxidative pentose phosphate pathway (PPP) in addition to glycolytic conversion to pyruvate [67]. Second, rather than a static process, the quantitative balance between shunting into the PPP versus proceeding through glycolysis changed with time over the first few days. Major functions of the PPP include assisting with maintenance of NADP/NADPH ratios and generating precursors for de novo nucleotide and lipid synthesis. TLR4-activated primary B cells showed substantial incorporation of [^14^C]-glucose into various lipid species and revealed that reduced capacity of the enzyme ATP-citrate lyase impaired in vitro differentiation [90], which is consistent with the identity of glucose as a meaningful source of membrane phospholipids but emphasizes a flow of carbon from glucose to acetyl-CoA and into phospholipids.

A recent stable isotope metabolic analysis provided evidence that most glucose was shunted into the oxidative PPP rather than being used for glycolysis in B cell blasts generated with anti-CD40 and IL-4 but no BAFF [91]. Glucose carbons were traced into ribonucleotides early after activation. Furthermore, a lipogenic precursor was detected in the mass spectra, indicative of glucose-derived lipogenesis during clonal expansion, perhaps involving the PPP. Overall, then, there was probably a substantial contribution of glucose to membrane biogenesis both through the PPP and glycolytic generation of acetyl-CoA. The fractional contributions—which may vary with time after activation or with different modes of mitogenic stimulation [67]—are less clear. Similarly, the extent to which different means of generating (PPP) or acquiring nucleosides contribute to the mass of RNA and DNA needed during doubling is not clear. Finally, evidence of mechanistic links among expression levels or stimulation of BAFF-R (a receptor for the stroma-derived cytokine BAFF), glucose uptake capacity, and the balance between tolerance and activation of B cells [71, 92] underscores potential pitfalls of culturing cells in the absence of BAFF.

Lymphoblasts increase their protein mass to prepare for doubling. Here, too, the relative paucity of quantitative data is notable. B lymphoblasts (LPS with BAFF) were found to have dramatically higher uptake of leucine than resting B cells [78] and similar increases in glutamine (Cho SH, unpublished observations). In that work, the focus was on the influx of a limited set of amino acids (leucine, glutamine, arginine, and lysine) at intracellular steady-state concentrations and on finding support for sustained activity of mechanistic target of rapamycin (mTOR) complex 1 (mTORC1) by mTORC1 derepression. Experiments involving limitation or deprivation of extracellular glutamine indicate that this conditionally essential amino acid supports B cell activation-induced proliferation [91, 93, 94]. Importantly, induction of *Aicda* mRNA, Ig class switching, and acquisition of GCB-like and PC-like phenotypic characteristics were reduced in a manner separable from division-linked processes in some studies [78, 79, 91, 93–95]. mTORC1 activity was also reduced by lowering the concentrations of all extracellular amino acids [95]. B cells haplodeficient for the essential Raptor subunit of mTORC1 recapitulated most aspects of B lymphoblast differentiation under glutamine or a.a. deprivation [78, 79, 95]. Because mTORC1 phosphorylates inactivating residues on ULK1 and thereby inhibits canonical autophagy, the high mTORC1 activity in activated and proliferating B cells might be expected to restrain this mechanism of protein and organelle turnover. However, semiquantitative assessments of canonical autophagy using imaging of LC3-GFP spots indicated that this process operated at similar levels in bulk FO and GC B cells [77]. AMP-activated kinase (AMPK)-catalyzed phosphorylation of activating residues on ULK1 is a driver of canonical autophagy. The capacity to increase noncanonical mechanisms of autophagy may, along with large increases in nutrient uptake and rapid cell divisions, bypass a need for the canonical process. Accordingly, the model can account for findings that both *Atg7* and the gene encoding the predominant AMPK in B cells, AMPKα1, were dispensable for B cell proliferation and for observed increases in the frequencies of antigen (nitrophenol (NP))-specific cells after immunization [83, 96].

### Energetics

Because metabolite flux depends on nutrient supply and uptake along with biosynthetic needs, the true nature of energetics and its regulation is more complex even than the “spaghetti-gram” of a Boehringer pathway chart. In part for this reason, attention—here and conventionally (e.g., [2])—has been given to three main pipelines for generating ATP (Fig. 2). These are (i) glucose utilization via glycolysis, either coupled to glucose oxidation in the Krebs (TCA) cycle or by aerobic glycolysis; (ii) anaplerotic conversion of glutamine for feeding of α-ketoglutarate (αKG, also termed 2-oxoglutarate or 2OG) into the Krebs (TCA) cycle [97, 98]; and (iii) FAO [98, 99]. Nonetheless, energy generation dependent on uptake from the external milieu—such as import of lactate or glucogenic amino acids such as alanine and aspartate and their conversion to pyruvate—remains a formal possibility for particular subsets or in certain milieux.

- Glucose equilibrates readily with the interstitium and circulates in blood at 3.5–5.5 mM (at the Km of the glucose transporter GLUT4, below that of GLUT1 and above that of GLUT3 [100]). Pioneering work [67], confirmed by others [68–71], documented substantially increased glucose uptake after mitogenic stimulation of B cells (BCR crosslinking with anti-IgM; TLR4 stimulation with LPS; CD40 crosslinking). An additional point emerged from these studies: the coupling of glycolysis to glucose oxidation (i.e., conversion of pyruvate to acetyl-CoA) is more efficient in B cells than in their T lymphocyte counterparts [70, 71]. Thus, although some increase in lactic acid secretion occurs, the magnitude of the increase in glycolysis substantially exceeds that of lactic acid secretion [70, 71]. Intravital labeling with 2-NBDG showed substantially increased fluorescence after type II T-independent stimulation of transgenic B cells with an NP-biased preimmune repertoire [87]. GC B cells represent one specialized class of B cells that, while diverse in relation to fate and cell cycle status, have an overall prevalence of cycling (S-phase) cells similar to that of in vitro blasts. Intravital labeling of these cells with 2-NBDG has reproducibly been found to be approximately double that of their naive counterparts [78, 87, 101]. (The concept of normalizing intake to putative cell size [87] to imply a lack of increase is noted only to comment that it is akin to believing that the gas consumption or carbon emissions of a large SUV are equivalent to those of a subcompact half its weight or size. A different, and important, issue is that of quantitating the relative demands for ATP generation in various cell states in the B lineage and even in subtypes of, say, GC B cells.) In light of published work in which the uptake of radiolabeled 2-deoxyglucose (2DG) (e.g., ^3^H- or ^18^F-2DG) differs substantially from that of the bulky analog 2-NBDG [88, 89], analyses with ^3^H-2DG are needed.
- Glutamine circulates in plasma at 0.5–0.7 mM, a range modestly below the Km for its uptake by lymphocytes [102]. Among its intracellular fates, this amino acid flows through a fuel line in which it is converted to glutamate by glutaminolysis, some of which feeds the generation of αKG [103, 104]. In this anaplerotic process, αKG enters the Krebs cycle for oxidative generation of ATP. Alternatives can include diversion to pyruvate or potentially diversion of citrate to acetyl-CoA that can be used for lipogenesis. Cancer cell and T cell use of glutamine to meet energy needs is well established (reviewed in [104–106]), but few published studies have explored the relative use of this pathway or the balance between glucose use and glutamine use in normal B cells. Anaplerotic consumption of glutamine likely makes substantial contributions [87, 107, 108]: it is instructive that in one study, in concanavalin A lymphoblasts (CD4 and CD8 T cells), only ~30% of glutamine carbons went to the TCA cycle, but these activated lymphocytes consumed over fourfold more glutamine than glucose in molar terms, putting glutamine on par with glucose in energetics [97].
  B cell blasts exhibit high basal respiration in glycolytic stress tests with the Seahorse metabolic flux analyzer (e.g., in [71, 77, 78, 87, 96, 101]). It is also instructive to compare glycolytic to mitochondrial stress test results (Brookens S, unpublished observations). The former test starts with a glucose- and fatty acid-free medium that contains glutamine, while the latter has glucose and pyruvate present. Basal oxygen consumption (respiration) of activated B cells and even acidification of the medium (oversimplified as representing lactate excretion) occur in the absence of glucose, pyruvate, serum, BSA, and fatty acids. One challenge in this area is that the concentration and nature of the adhesive (Cell-Tak vs. poly-D-lysine) have major effects on Seahorse data outputs (Raybuck AL, Boothby M, unpublished observations). Moreover, a fundamental aspect of flux in metabolic pathways is the conditional nature of the degree to which a particular nutrient or pathway is used. The results depend greatly on assay conditions (noted already in [98, 103]) that in turn differ from conditions in the interstitium in vivo. These concerns notwithstanding, it is likely that glutaminolysis contributes substantially to energy generation in B lymphoblasts. Such a function could either be part of, or in addition to, the dependence of proliferation and differentiation on sufficient concentrations or masses of glutamine in the environment of a mitogenically stimulated B cell in vitro.
- Fatty acid oxidation is another source of energy. Concentrations of fatty acids in serum are vastly more variable across a population (10- to 30-fold differences in a sampling of healthy adults [109]) than those of amino acids and glucose, for which even twofold differences are pathological. Longer-chain fatty acids are bound to serum proteins such as albumin to supply the interstitia of lymphoid organs and B cells within them. A recent work analyzed B cells after T-independent (NP-Ficoll) activation or immunization with haptenated protein to elicit GC B cells using a model built on transfers of NP-reactive B1-8i, Vκ–/– B cells into mice whose B cells could not bind the hapten [87]. This work provided evidence indicating that a substantial fraction of respiration by the activated B cells in Seahorse analyses was sensitive to chemical inhibitors of FAO during the analyses, which presumably involved fatty acids from bovine serum albumin. For resting (naive) B cells, approximately 60% of respiration was sensitive to etomoxir, which is commonly used to inhibit mitochondrial FAO. In addition, substantially increased levels of a peroxisomal protein, PMP70, were found in the B cells activated by T-independent immunization, and chemical inhibition of respiration with thioridazine pointed to FAO by this organelle. Taken together with evidence of increased staining with fluorophore-labeled palmitate and ^13^C-palmitate tracing results, the findings indicated that FAO contributed substantially to the energy requirements of these activated B cells.

### ROS, redox, and signaling downstream of nutrient- and energy-sensing pathways

The electron transport chain (ETC) intrinsically generates reactive oxygen species (ROS), so increased flux through the TCA cycle and heightened respiration highlight the potential functions of ROS in modulating the decisions of B cells after activation. The aggregate literature highlights the need for better and more quantitative tools along with the importance of compartmentalization (e.g., cytosol vs. mitochondria). The net production and impact outside the mitochondria (or peroxisomes) depends both on the rate of generation—for instance, by electron transport complexes I and III—and on the capacity to resolve ROS. This latter step intersects with glucose and glutamine metabolism, since glutamate is essential for glutathione synthesis and since the oxidative PPP generates NADPH to reduce oxidized glutathione. Reactive oxygen can inhibit the activity of PTEN, a lipid phosphatase that lowers steady-state levels of PIP3 via lipid kinase phosphatidyl-inositol 3-kinase (PI3K). Accordingly, it has been hypothesized that BCR-induced ROS generation can increase the activity of signaling enzymes downstream in the PI3K pathways [110].

Several papers have provided evidence in favor of this model but also highlighted that BCR crosslinking leads to sustained ROS in at least two mechanistically distinct phases [111, 112]. For the first few hours, increases in ROS production and levels elicited by BCR stimulation appear to be mediated by proteins of the NADP oxidase complex, e.g., *Nox2* (or *Duox2* in a paper on TCR-induced ROS) [112, 113]. This initial phase appears to increase mitochondrial respiration, which can then sustain the increased PIP3 or mitigate the activity of the protein tyrosine phosphatase SHP-1. As noted in [112], conflicting results suggest that ROS instead can decrease B cell activation and antibody responses [114, 115]. The results of cell line experiments reporting interplay between BCR-induced ROS generation and the calcium signaling pathway [116] highlight complexities that are likely due to the spectrum of target molecules (e.g., all those with reactive cysteines that might be modified by ROS). Consistent with this possibility, ROS induced by B cell activation reportedly inhibit the protein tyrosine phosphatase SHP-1, which limits the strength of BCR and other types of signaling in amplitude and time [111]. One line of in vitro evidence suggested a model in which tuning of the relative mitochondrial mass and rate of ROS generation may influence the balance of differentiation outcomes [117]. An analysis of activated B cells provided alternative evidence of distinct subpopulations defined by flow cytometric qualities that included mitochondrial ROS [118]. Of note, the differences were reported to have both predictive value in terms of an inverse relationship between the likelihood of yielding switched (IgG1^+^) B cells or plasma cells and an impact on the activity of an enzyme involved in heme biosynthesis [118].

Examples in other biological settings indicate that the modulation of reactive cysteines in signaling enzymes will likely involve more targets than those noted here [119]. Of note, modes of protection against oxidative damage likely influence different branches of B cell responses that lead to Abs. The requirements of B2 B cells were found to differ from those of B1 or MZ B cells [120], and the same loss-of-function mutation had a more markedly nonredundant effect on T cells [121]. Accordingly, glutathione peroxidase-related findings underscore cell type and stage specificity, perhaps due to increased redundancy in the glutathione and thioredoxin systems of FO B cells and PCs (elegantly reviewed in [122, 123]). To what extent the capacity to implement glutamine-dependent synthesis of glutathione regulates progression of activated B cells—akin to T cells [124, 125]—or to restore its reduced state via PPP activity are important questions to answer for elucidation of metabolic regulation in the B lineage.

As noted above, activation of PI3K results from BCR stimulation is likely maintained in part by the actions of ROS, and is both enhanced and prolonged by costimulation of B cells via CD40, TLR, and other receptors (reviewed in [1, 2]). Two major signaling complexes downstream of PI3K share the serine-threonine kinases mTOR, mTORC1 and mTORC2. Both complexes are inhibited by rapamycin treatment of lymphocytes, albeit more slowly for mTORC2 (hours) than mTORC1 (minutes) [126–128]. Inhibition of both mTORC1 and mTORC2 and their ubiquity prompt caution when teasing out their respective contributions to Ab responses or plasma cells, as do issues of secondary effects and adaptation after irreversible inactivation of pathways whose activity likely changes over the time course (days) of the progression from naive B cells to Ab-secreting cells. In addition, further caution is warranted because although mTORC1 and mTORC2 are major (but not the only) effectors of the signal initiated by PIP3, some results with altered PI3K appear opposite to those with mTOR (discussed below [129–132]). Induced deletion of *Rictor*, which encodes an essential subunit of mTORC2, led to a substantial defect in B cell proliferation attributable to both decreased cell cycling and reduced survival signaling [133]. BCR stimulation of Rictor-deficient B cells led to increased expression of pro-apoptotic BH3-only members of the Bcl2 gene superfamily (e.g., Bim) and a failure of the normal induction of survival gene expression (*Mcl1*; *Bcl2l1*) [133, 134]. Unlike after inactivation of mTORC1, however, no effect on Ig class switching was apparent. Recent work with *Cd19*-Cre-driven deletion of the essential SIN1 subunit of this signaling complex confirmed a requirement for mTORC2 in proliferation and antibody responses [135]. SIN1-depleted B cells exhibited lower respiration and extracellular acidification in both the resting and anti-IgM-treated states, along with lower steady-state levels of the c-Myc protein. These findings suggest that, as in other cell types, mTORC2 influences intermediary metabolism in B cells.

Similar to mTORC2, mTORC1 supports B cell proliferation [78, 79, 94, 136]. Such a function is consistent with a general view of mTORC1 as mediating coordinated enhancement of anabolism. Regardless of the precise molecular functions of mTORC1 in B cells in vivo, there is at least one unresolved difference in the data. In some cases, interference did not lead to a global or generic defect in B cell activation or progression to antibody secretion: the concentrations of immunization-induced antigen-specific IgM elicited in B cell type-specific *Rptor* Δ/Δ mice or mice with rapamycin treatment were higher than those in controls [78, 79, 137]. Alternatively, decreased antigen-specific IgM and defects in antibody secretion have been noted [138, 139]. The nutrient-regulated complex has reproducibly been reported to mediate heightened expression of AID, the enzyme that effects class switch recombination and SHM [78, 79, 94]. This effect on AID is manifested in decreased switching, in vivo and in vitro [78, 79, 94], and in a lower frequency of somatic mutations in an anti-NP response [79]. An apparent conundrum worth noting relates to PI3K and mTORC1 in class switching. Analyses of increased PI3K activity (either via a gain-of-function mutation or elimination of PTEN from B cells) as well as selective chemical inhibition of the p110δ catalytic isoform have shown that overall, PI3K tends to inhibit Ig class switching [129–132]. While the mechanisms for this disparity are not fully resolved, inhibition of AKT completely reversed the PI3K-driven suppression of switching in one study [131]. Thus, excessive function of AKT—which is primarily activated by PIP3-dependent T308 phosphorylation, operates independently from mTORC1, and is only modulated in terms of activity by mTORC2—likely dominates over mTORC1 as a regulator of switching.

In further analyses, mTORC1 activity in activated B cells in vitro was needed to maximize glucose-stimulated extracellular acidification (semantically simplified to “glycolysis,” although glucose-stimulated extracellular acidification can differ substantially from the actual glycolytic rate). Moreover, inhibition of glycolysis with 2DG reduced *Aicda* gene expression and AID protein levels [79]. Interference with glycolytic flux in vitro also attenuated the induction of *Irf4* and *Bcl6* gene expression [79], implying that glucose flux may regulate plasma cell differentiation. While these findings suggest that glycolytic flux regulates gene expression, specific molecules mediating such effects remain to be determined. In addition to the mechanism by which ROS help to sustain heightened mTORC1 activity, this complex may contribute to a positive feedback loop in which it increases the mRNA levels of amino acid transporters such as LAT1 (*Slc7a5*) and ASCT2 (*Slc1a5*) [78, 79], which mediate entry of leucine and glutamine, respectively. Finally, while complete loss-of-function mutations are informative, they often will be more extreme than the physiological range within which dynamic regulation operates. In this regard, findings of reduced B cell proliferation and switching to IgG2c in the setting of Raptor haplodeficiency suggest that activity of the complex within a range regulated by either a.a. deficiency or hypoxia [78, 79] is physiologically relevant.

Another documented mTORC1 function is to promote mRNA translation efficiency, including during B cell ontogeny [140–142]. Consistent with this, careful titrations allowed identification of a mechanism in which mTORC1 inhibition reduced translation of *Aicda* mRNA into AID protein in B cells via mTORC1 phosphorylation of eIF4 binding protein (4EBP-1) and eukaryotic (translation) initiation factor (eIF)4E [94]. A recent work on B cells reported that stearoyl-CoA desaturase, a component of biosynthetic pathways that catalyzes desaturation to generate monounsaturated fatty acids (MUFAs), was downstream of mTORC1 [143]. Supply of MUFAs was essential for B cells during rapid proliferation, but uptake and utilization of these macromolecules from the interstitial spaces appeared to suffice in the absence of synthesis within the B cells. This work provides direct evidence of fatty acid uptake into B lymphoblasts and an essential need for uptake of this nutrient.

The highly conserved AMP-activated serine-threonine kinase AMPK exhibits a function opposite to the general promotion of anabolism by mTORC1. AMPK maintains cellular energy homeostasis by promoting catabolic pathways in energetically stressed conditions such as nutrient-limited environments [144], e.g., glucose limitation for B cells in vitro [96]. Among the scores of targets, AMPK catalyzes several protein phosphorylation events that reduce mTORC1 activity [145], e.g., phosphorylation of the TSC2 tumor suppressor and of the core mTORC1 subunit Raptor. In B cells, one isoform of the conventional AMPK, AMPKα1, appears to account for essentially all detectable modifications of substrate proteins that have been analyzed, such as inhibitory phosphorylation of acetyl-CoA carboxylase, an enzyme central to fatty acid biosynthesis (reviewed in [145, 146]). Moreover, mTORC1 activity was increased in AMPK-deficient B cells analyzed directly ex vivo [96]. Among intriguing findings, AMPK has consistently appeared to be dispensable for the induction of B cell activation markers as well as for the formation of steady-state B cell populations in primary GCs ([96, 147, 148]; reviewed in [149]). Although p-AMPKα1^T172^ expression increases with activation [148], anabolic pathways presumably dominate B cell metabolism during activation and proliferation. Interestingly, the kinetics for p-AMPKα1^T172^ expression after activation parallel the deceleration of biomass accumulation in activated B cells, indicating that p-AMPKα1^T172^ may play a role in dampening unlimited cell growth through negative regulation of mTORC1 and other anabolic substrates during activation [148].

## Decisions, decisions—B cells after activation

As summarized in the section “Introduction and overview,” activated B cells and their progeny can each adopt one of several potential fates. Some of these states represent irrevocable “decisions” or “choices,” most extremely deletion or death, but also the decision to end up as a plasma cell fully committed to antibody secretion. MZ B cells can shuttle rapidly to a B cell follicle. Apart from death, activated B cells can resume quiescence as memory B cells, proceed directly to the plasmablast-plasma cell pathway, or participate in a GC reaction in a secondary follicle. Once in a GC, the immigrant and its progeny again are partitioned among fates—death, quiescent survival in a memory pool, continued cycling in the GC, or progression to plasmablast/plasma cell states. A key issue is to elucidate the influences of nutrient supply and programming of intermediary metabolism on these outcomes. A corresponding issue is the extreme paucity of data on the variance in nutrients or the sufficiency of their supply in different parts of the microanatomy, especially during normal physiology after immune challenge.

### Signaling and nutrient-sensitive mechanisms

**Fig. 3:**
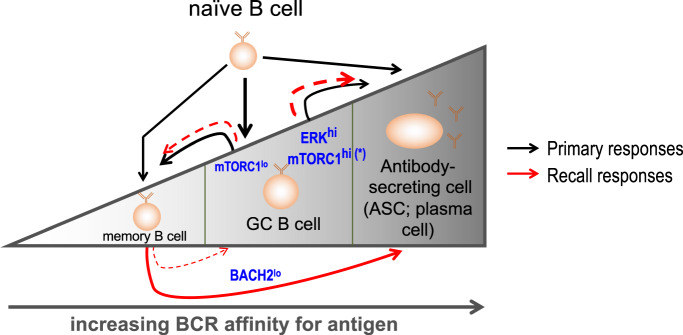
Summary of relationships and potential connections of BCR affinity, selected signals, and fate choices for B cells. As reviewed in the main text, the likelihood that a naive B cell, after its activation, flows into the memory pool, stably participates in a germinal center reaction, or undergoes extrafollicular differentiation directly to a plasma cell fate is influenced by BCR affinity (or avidity) for the antigen (indicated by the triangle and indicator arrow below it). GC B cells also contribute to the overall memory pool, generally after some degree of affinity maturation (not captured in this cartoon). Aspects of the relationship to signaling via ERK and mTORC1 activity are not fully established or settled, e.g., that high mTORC1 activity fosters increased PC differentiation among GC B cells. As discussed in the text, however, ERK^hi^ and mTORC1^hi^ cells appear to be favored for progression toward the PC fate, but whether BCR engagement by higher- versus lower-affinity ligands (antigens) causes heightened ERK or mTORC1 activity is not clear. For the memory pool, which will tend toward a more somatically mutated and selected BCR repertoire, memory cell activation will favor PC differentiation among BACH2^lo^ MBCs, but some activated memory cells do enter a new GC reaction, which in turn can yield new MBCs and ASCs

One fate choice for activated B2 B cells is “whether to GC or PC;” the affinity of the protein antigen-BCR interaction is a major determinant of this choice ([30, 58]; reviewed in [29, 32]). Recent work provides evidence that affinity and avidity also partition B cells between an early memory fate versus one in which they stay in the GC [55, 56] (Fig. 3). BCR affinity for antigen is interwoven with the elicitation of help (e.g., CD40L and cytokines such as IL-4 and IL-21), as B cells capture, internalize, process, and present helper-specific epitopes on their MHC-II molecules (reviewed in [33, 34]). Whether and how the affinity and duration of interaction lead to differences in metabolic programming or function are not clear. In foundational work using cloned T cells and altered peptide ligands, antigen-TCR interactions influenced rates of extracellular acidification [150]. Extracellular signal-regulated kinases (ERKs) probably signal at different intensities depending on the peptide-MHC complex [151–154] and can alter glucose, glycolytic, and glutamine uptake and metabolism in T cells [155, 156]. Accordingly, it may be that ERK activity regulates not only the differentiation of T cells into different effector subsets but also the metabolic programming vital for the differentiation and function of CD4 and CD8 T cells [105]. Consistent with the potential for ERK titration to alter B cell physiology and differentiation, both BAFF-R and BCR stimulate this MAP kinase, which in turn regulates the progression of human and mouse B cells to antibody-secreting plasma cell states [157–162]. Moreover, interference with the conversion of diacylglycerol to phosphatidic acid, as catalyzed by diacylglycerol kinase, increased the sensitivity to IgM crosslinking, the amplitude of ERK activation, and the generation of antibody-secreting cells after immunization in one study [163]. Moreover, ERK signaling can promote Blimp1 expression and PC formation [160, 162]. Collectively, these papers suggest a model akin to that suggested by findings in CD4^+^ T cells [153] in which quantitative aspects of ERK activity may guide the distribution of daughter B cells among different fates. BCR transgenic systems could address these questions for B cells, but we are unaware of papers directly testing a model in which quantitative increases in ERK drive metabolism downstream from higher-affinity Ag-BCR engagement to push B cells toward a PC fate (Fig. 3).

An additional issue involves the TNF and TNF receptor superfamily pair of BAFF (BLys) and BAFF-R, which are key regulators of B cell activation potential and survival. This pair has been reported to induce ERK as well as mTORC1, PKC-β, and AKT and to increase glycolytic metabolism of B cells via a mechanism involving at least the two latter signaling kinases [92, 159, 161]. In addition to underscoring the importance of integrating BAFF/BAFF-R into in vitro analyses, these papers suggest that BAFF-induced ERK may contribute to the in vivo function of ERK and metabolic flux in the generation of Ab-secreting cells Fig. 3. It is possible that the inclusion or omission of BAFF in cultures of B cells in vitro is pertinent to the assessment of some quantitative or qualitative differences among the data from different groups using different approaches. Nonetheless, most researchers and papers have appeared not to use recombinant BAFF in concert with mitogenic signals.

Some cycling B lymphoblasts collaborate effectively with cognate helper T cells and enter secondary follicles, i.e., GCs that start to form approximately 3.5 days after immunization. This “choice” is an important one, but limitations in the tools to study this step mean that analyses of any potential link to nutrients, nutrient sensor systems, or metabolism are currently not feasible. Studies that have scored GCs or used hapten NP for Ag-specific GC B cells have found that GCs were unaffected by a lack of AMPK in primary Ab responses [96, 147, 148]. Moreover, no defect was detected in screenings for affinity maturation [96, 148]—a process amplified by GC reactions although not absolutely dependent on them [43, 164]. These findings suggest that although B cells may be competing for limited nutrients to support the demands placed on them in the average GC, they may normally avoid any long period of energy stress manifested as increased AMP [91, 96]. These findings raise unanswered but intriguing questions relating to quantitative features of the impact of increased mTORC1 on GCs and their outputs. Hyperactivation of mTORC1, by either loss of AMPK or loss of tuberous sclerosis complex 1 (TSC1) protein, did not affect GC formation ([91, 96, 148, 165]; reviewed in [149]). Alternatively, hyperactivation of mTORC1 signaling by TSC1 elimination or by a constitutively active mutant of RagA (a GTPase that participates in mTORC1 activation) caused GC B cell retention in the dark zone (DZ) and impaired affinity maturation [136]. The intensity of mTORC1 signaling appears to have consequences on differentiation. Perhaps for reasons mechanistically distinct from increased mTORC1, a lack of AMPK in B cells led to an initial increase in memory-phenotype B cells [96], whereas GC B cells with relatively lower mTORC1 activity tended to favor a memory B cell fate [54]. Taken together, these findings suggest that mTORC1 is tuned to an optimal level of activity so that at one level of increase (AMPKα1 loss), there is no increase in the GC, whereas GC fitness and outputs are reduced at higher levels with loss of *Tsc1* or a gain-of-function RagA mutation (which may influence B cell development). The possibility that the mTORC1 data tie in with recent work indicating that the source of GC-derived memory B cells is a preimmune pool with BCR of lower affinity or avidity for antigens is attractive but, as with ERK, it will need to be tested. This synthesis provides a model, and integrated work that quantifies the increase in mTORC1 activity is not available.

Rapamycin treatment dramatically reduced the numbers of GCs in mouse studies [137, 138] (section “ROS, redox, and signaling downstream of nutrient- and energy-sensing pathways” above), but the interpretation of such results is complicated by the effects of the drug and the requirement for mTORC1 and mTORC2 in Tfh cells [127, 128, 166, 167]. An elegant approach using acute pharmaceutical inhibition of B cells transferred into a mouse line engineered with a rapamycin-insensitive mTOR mutant provided evidence that mTOR is crucial for GC B cell shuttling and partitioning between light and DZs [136], but how this impacts plasma cell and antibody outputs has not been established. Parallel work used genetic approaches that involved conditional inactivation of the Raptor-encoding gene *Rptor* and found that a lack of mTORC1 caused substantial reductions in GC B cells that were especially marked for the NP-specific population and reduced both SHM and high-affinity IgG1 (to ~0.5× and ~0.05× normal levels, respectively) [79]. Although it has not been confirmed by what is currently the most powerful means of testing GC-restricted loss-of-function for mTORC1 (e.g., use of the *S1pr2*-CreERT2 transgene for GC-restricted inactivation of alleles [53–55, 168]), the evidence to date suggests that this signaling node regulates dynamics and outputs in the GC.

mTORC1 activity is regulated by PI3K but also by reversal of repressor influences when a cell senses sufficiency of key nutrients that license an anabolic program ([169–173]; reviewed in [174, 175]). A multitude of cell surface receptors—including BCR and CD40 stimulated by cognate Tfh cells—can initiate PI3K signaling, and coinhibitory receptors may act in part by inhibiting this protean pathway. Accordingly, it is difficult conclusively to link particular aspects or phases of activity with one or another of these receptors based on current evidence. That said, expression of a fluorophore-encoding allele tracking expression of the proto-oncogene protein c-Myc, which coordinates increased uptake and utilization of glucose and glutamine in B lymphoma and primary T cells [107, 176, 177], has been tied to PI3K but found to be transient and restricted to a subset of GC B cells [178, 179]. An analysis of single cells showed that P-ERK, P-PLC-γ, and a phosphoprotein that particularly marks B cells as having undergone recent BCR stimulation (P-BLNK) tended to occur concurrently in GC B cells along with increased c-Myc [101]. mTORC1 can mediate c-Myc induction by enhancing its translation [140–142], which, like that of BCL6, can be uncoupled from mRNA levels. This regulatory function mediated by S6 kinase downstream of mTORC1 might lead to the expectation that S6 phosphorylation is increased in the Myc^+^/^hi^ population of GC B cells. Instead, these traits (P-S6^hi^ vs. c-Myc^hi^) were found to be mostly nonoverlapping except for a high congruence in some IgG1^+^ GC B cells [101]. A difference between IgM and IgG1 BCR-induced signal transduction [180–182] might account for these findings, but confirmation via challenging experiments will be required to test this speculative model. Similarly, some of the separation of P-S6^hi^ versus Myc^hi^ cells may be due to the capacity of the transcription factor AP4, whose expression is c-Myc- and then IL-21-dependent, to sustain metabolic programs in addition to GC B cell proliferation [183]. In any event, these findings obtained through application of a powerful single-cell (phospho-)protein scoring approach point to a crucial consideration regarding the programming of GC B cells: these cells are diverse and dynamic, so monolithic interpretations should be avoided [184–186].

Most GC B cells are fated to die and may have different characteristics from those that have been positively selected. The partitioning of the GC creates a set of DZ B cells that proliferate, are licensed for AID-mediated mutation, and accordingly are subject to p53 induction [52]—which influences cellular metabolism [187, 188]. In contrast, DZ survivors that move to the LZ are thought to be arrested in the cell cycle ([51, 136]; reviewed in [33, 34]) and to be able to successfully compete for a fresh input of help (e.g., CD40L) in order to resume proliferative population growth. In addition, a given GC will contain a mixture of B cells that either will continue iterative DZ-LZ shuttling or continue on their way toward quiescence as memory B cells or as plasma cells [53, 54, 58]. Nonetheless, the current data support the functions of transcription factors such as c-Myc, perhaps downstream of mTOR; AP4 downstream of c-Myc; and FoxO1. The involvement of FoxO1, likely downstream of PI3K [189, 190], implies that mTORC2 activity—which appears essential for GC organization [133, 135]—mediates signaling between the lipid kinase PI3K and transcription factors.

In addition to FoxO1, GSK3 is another solidly established target of mTORC2, which places an inhibitory phosphate on the regulatory S9 residue of GSK3. In a study involving multiparameter investigation of phosphoproteins, a major subset of P-GSK3(S9)^hi^ cells overlapped with the cells with the strongest signals, which provided evidence of recent BCR engagement [101]. Of note, different means of achieving dual inactivation of the *Gsk3a* and *Gsk3b* genes (i.e., use of Cγ1-Cre or the tamoxifen-activated B cell-specific CreER) have yielded converging findings of reductions in GC B cell populations [101, 191]. While these data indicate that GSK3 is a key contributor to the GC, the disparity between data obtained after mTORC2 versus GSK3 inactivation raises the possibility that an input to GSK3 other than PI3K—such as an input from the WNT pathway [192]—is a more important physiological factor than PI3K signaling. Another apparent paradox lies in evidence that GSK3β phosphorylation of Mcl-1 in T cells destabilizes this anti-apoptotic Bcl2 family member [193, 194], given the essential role of Mcl-1 in GC B cells [195], as GSK3-deficient GC B cells would be expected to have increased Mcl-1 levels. Notwithstanding the untested but important possibilities, studies on GSK3 inactivation point to an important function in directing fate potential for GC B cells.

One “binary choice” is memory versus continuation in the GC. Recent work suggests that a bifurcation of memory versus GC continuation exists based on BCR affinity for antigen [55, 56]. In one iteration, there is a fundamental difference between the original germline-encoded receptor leading to each fate, such that memory-fated GC B cells start out with lower BCR affinity and avidity for antigen than those that persist in LZ/DZ cycling [55]. In addition, such affinity determinism appears to distinguish cells that will become plasmablasts, renew proliferation in the DZ, or move toward quiescence as memory cells [56]. This raises the possibility, yet to be confirmed, that signal intensity or activity along the PI3K or mTORC1 pathways may be reduced. Potentially consistent with this model, memory precursors have been reported to have reduced mTORC1 activity [54]. In the work noted above, however, increased mTORC1 enhanced the initial output of memory-phenotype B cells [96]. It may be that barcoding approaches will be needed to tease out how much given phenotypic subsets of GC B cells derive from a single precursor of set BCR affinity. In addition, the difficulties in quantitating exact degrees of increases or decreases in the activity and localization of mTOR complexes pose a major challenge to resolution of differences among papers on the relationship between mTORC1 and GCs or their outputs (reviewed in [149] and, for T cells [196]). Limiting dilution analyses to approximate the fate potential for single naive B cells have underscored that there is a range of options influenced but not rigidly determined by BCR affinity, such that many B cells can assume any of the potential fates [197]. At present, therefore, it remains likely that a single low-/intermediate-affinity B cell in the GC can either become a memory B or, perhaps after a round of proliferation and SHM, yield plasma cells. Important insights, however, have emerged from single-cell transcriptomics coupled with BCR mutation analyses [198]. This work provides strong evidence that oxidative phosphorylation rates are higher in the subset of positively selected GC B cells than in other subsets of secondary follicles. Importantly, *Aicda*-driven inactivation of Cox10, the gene encoding a component of mitochondrial ETC complex IV, led to decreased proliferation and steady-state numbers of GC (but not other) B cells and culminated in reduced outputs of antigen-specific PCs [198]. These results indicate that optimal GC conditions depend on mitochondrial ETC function in B cells that activates AID expression after immunization, probably in the GC B cells themselves.

In any event, elevated PI3K and mTORC1 activity appears to increase the likelihood of an activated B cell (GC or extrafollicular) differentiating toward the PC fate [199, 200]. CD19 may be a central hub facilitating the initiation of signal transduction along these pathways [201], but in any case, involvement of Ras-like GTPases such as R-Ras2 [202] and conventional recruitment of p110δ, a p110 catalytic subunit of PI3K, increase the generation of phosphatidylinositol-(3, 4, 5)-triphosphate (PIP3, or PtdIns(3, 4, 5) P3) from PI (3, 4) biphosphate (PIP2) ([203]; reviewed in [204]). As discussed above, ROS generated by normal metabolism of B cells may sustain such increases by interfering with the catalytic activity of the lipid phosphatase PTEN. The importance of sustained PIP3 in promoting progression toward plasma cell differentiation has been underscored by work in which this process was modulated by enhancing or reducing levels of PI3K products via PTEN regulation or a mutated adapter protein [205–208]. Conversely, signals from Syk [209], Cbl-mediated degradation [210], and GSK3 are reported to restrain the progression of GC B cells to plasmablast/plasma cell fates, while sufficient ERK activity appears to reduce the levels of the BACH2 protein, which antagonizes the ability of Blimp1 to drive plasma cell differentiation [162, 211].

The activity of PI3K and mTORC1 influences mitochondrial mass and function through regulation of mitochondrial genesis and quality control through mitophagy. Emerging work has used an elegant approach to directly intervene in mitochondrial replication and function in B cells through the expression of a mutated helicase [212]. This study provides further [198] evidence that maintenance of sufficient mitochondrial mass and function is essential for achieving normal GC B cells and class-switched antibody responses. Consistent with mTORC1 inhibition of canonical autophagy (including mitophagy), GC B cells and the initial formation of humoral memory (B and plasma cells) were maintained normally despite elimination of essential components of the conventional autophagy mechanism (*Atg7* and *Atg5*, respectively) [213, 214]. Along with the finding that elimination of AMPK-catalyzed phosphorylation of a key activating site on ULK1 failed to decrease the generation of memory B cells or plasma cells [96], the evidence indicates that canonical autophagy is not needed for the emergence of these cell types from the GC reaction, whereas noncanonical autophagy may be more crucial at this stage [77].

### Nutrient supply, uptake, and usage

Ultimately, of course, the signaling and gene expression pathways (section “Signaling and nutrient-sensitive mechanisms”) yield different programs of nutrient uptake and intermediary metabolism in the varied subsets of B cells within the GC. Reciprocally, nutrient supply and uptake are vital regulators of mTORC1 activity in addition to receptor-induced PI3K or lysophosphatidic acid as initiators of mTOR signaling (reviewed above). For activated B cells, the programs can be divided between energy generation and synthetic processes (anabolism). The nutrients that have been analyzed are glucose, glutamine, and long-chain fatty acids (LCFAs). Metabolism partitions each nutrient between energy generation and anabolic function and—as known for lymphocytes since the 1980s—flux in use of one nutrient will be affected by supply of the others [99]. Another key gap is that it is not known what the anabolic costs are as a percentage of the overall ATP needs—notably, the maintenance of ion gradients across the plasma membrane—and how much these other parts of the budget change after activation or differentiation.

For T cells in a number of pathological settings (cancer, pulmonary tuberculosis), many papers provide evidence of amino acid or oxygen depletion and even improved function with supplementation (e.g., [215–217]). Might this concept apply in humoral immunity and directly to B cells? A pair of reciprocal questions follows from that relationship. (i) Is nutrient delivery to the interstitial fluids so robust that in follicles or in the peritoneum (for B1 B cells), there is no potential for improving outputs—for instance, of the number or longevity of plasma cells after immunization? (ii) In various forms of malnutrition, do any B cell-intrinsic deficits of performance arise due to nutrient insufficiency? In general, there is a large body of literature relating to the second question; however, almost none is specific to B lineage cells, let alone GC B cells or LLPCs in their microenvironmental niches. Although not definitive, several published examples suggest that shortfalls in local nutrient delivery do affect the progression to Ab-secreting cells and can reduce humoral protection.

- Glucose: relatively little stage-specific insight is available even though (or because) glucose use—especially via glycolysis—was a focus of the early exploration of the interplay between metabolism and function in this lineage. Glucose concentrations in interstitial fluids are so critical that only in extreme circumstances might they fall to a level below what would be needed for cells under conditions of normal immune physiology. GC-phenotype B cells take up approximately twofold more of the intravital probe 2-NBDG, a bulky glucose analog, than their naive B precursors [78, 87, 101, 218]. To what extent is glucose import or intracellular flux vital for the formation, maintenance, or output of GC B cells? Treatment of immunized mice with 2DG collapsed the GC B cell population [101], but as noted [87], the data indicating that Tfh cells require glucose import and flux [166] raise the possibility that the effect of 2DG on GC B cells was indirect. B cells express at least three facilitative transporters of glucose. An important but preliminary work generated B lineage-restricted loss of function for the GLUT1 transporter using *Cd19*-Cre [71]. This approach led to reductions in the establishment or maintenance of the overall B cell population and, early after immunization, decreases in both total and NP-specific antibodies similar in magnitude to the developmental defect [71]. However, the developmental effect along with the need for information on the clonal expansion of antigen-specific B cells and on counts of plasma and GC B cells leaves it unclear at which stages along the B lineage sufficient glucose flux is essential for basal antibodies (largely stemming from B1 and MZ B cells) and antigen-elicited humoral responses.
  The possibility that intracellular glucose flux and sufficient glucose oxidation are essential for the normal efficiency of GC B cell development was also supported by an analysis of this process using an in vitro model system driven by CD40L and BAFF [218]. In this latter work, IL-4-stimulated increases in glucose uptake and mitochondrial oxidative metabolism akin to those in earlier studies [69–71] were tied to increased αKG, which was important for increased *Bcl6* mRNA expression and in vitro production of iGB cells (i.e., CD40- and BAFF-stimulated cells proposed as proxies for GC B cells) [218]. However, apart from differences between the model system and GC B cells in vivo, the analysis did not distinguish glucose oxidation after glycolysis from anaplerotic generation of αKG from glutamine. Glucose contributes to the energy needs of dividing cells. A recent paper concluded that glucose makes minimal contributions to ATP generation in a particular system of purified ex vivo GC B cells [87] based on Seahorse metabolic flux data, the minimal ECAR (which is dependent on the balance between LDH and pyruvate oxidation), and ^13^C-glucose tracing. However, bioinformatic analyses of the RNA-Seq profiles of the naive and GC B cells used in that work—which involved transfers of anti-NP BCR knock-in cells into a nonphysiological recipient setting—suggested that these cells differed from those in a less-engineered polyclonal setting and response [186]. Moreover, the magnitude of the glucose-induced reduction in respiration for the anti-NP GC B cells was comparable to their fatty acid-dependent respiration [87], which is consistent with substantial glycolytic ATP production. Accordingly, important questions about glucose use in B cells as a part of normal GC physiology remain open—both for energetics and for other vital purposes such as redox regulation and provision of precursors for nucleic acids and glycosylation through the PPP.
- Glutamine: as discussed above, limitation of glutamine in vitro decreases B cell proliferation [91, 93, 94, 97]. In a recent work, a strong wave of plasmablast differentiation in response to Plasmodium infection in mice caused a decrease in the humoral response and in the frequencies of GC Tfh and B cells [219]. Provision of extra glutamine in the drinking water mitigated these negative effects, enlarging the GC in infected mice and increasing the impaired memory output. However, several key questions were not answered by this work. Whether GC B cells were direct beneficiaries of glutamine as opposed to Tfhs, Tregs (e.g., [220, 221]) or other pertinent cells in the model and the changes in interstitial glutamine with Plasmodium infection or glutamine supplementation (e.g., in the white pulp or the follicle) remain to be determined. The actual impact on FO concentrations was not measured (especially due to use of spleen tissue, with its admixture of red pulp and other structural features), and the systemic effects of enteric glutamine supplementation can render interpretations or mechanisms quite complex. Moreover, it is unclear for cells in the GC whether a glutamine (or glucose) requirement is a matter of energetics or instead links to biosynthetic needs such as the supply of uridine diphosphate *N*-acetylglucosamine (UDP-GlcNAc) to support intracellular glycosylation [222]. Nonetheless, the work provides an important line of evidence supporting the potential for local nutrient depletion (in this case by plasmablasts) to affect immune outputs.
- Fatty acid oxidation: exogenous fatty acids in the circulation, although widely variable in their concentrations and profiles among individuals, are important in cell physiology. Functional roles for at least two distinct forms—long- and short-chain fatty acids (LCFAs and SCFAs, respectively)—have been studied. SCFAs (e.g., acetate, butyrate, and propionate) have long been known to potentially feed into pathways of posttranslational histone modifications that affect rates of gene transcription. Interest in their systemic effects in normal physiology has been stimulated by the recognition that the generation of these SCFAs by gut microbial processing of dietary components—for instance, the fiber components in food—reflects a capacity for an altered diet or gut microbiota to change circulating SCFAs. Recent work provides evidence that such processes can modulate the qualities of antibody responses via B cell-intrinsic effects on fates of activated B cells [223, 224].
  An early work on unfractionated lymphocytes quantified the effects of an LCFA (oleate) and SCFA on resting and activated lymphocytes in vitro [99]. As noted above, naive B cells exhibited substantial rates of long-chain FAO in assays using tritiated palmitate [70]. A system engineered to enhance yields of NP-binding B cells was used to directly test GC B cells in comparison to naive and NP-Ficoll-activated counterparts [87]. Three salient findings were that (i) respiration (oxygen consumption, i.e., OCR) by the GC B cells was approximately 1/4 that of the in vivo NP-Ficoll-activated population, (ii) the fraction of OCR sensitive to a compound that inhibits mitochondrial FAO was comparable to what was observed for the other two ex vivo populations (naive and NP-Ficoll-activated), and (iii) the rate of respiration imputable to FAO was comparable to the basal oxygen consumption of the purified GC B cells when only glucose, glutamine, and pyruvate were used. Pharmacological interventions using inhibitors provided evidence that, as with the NP-Ficoll-activated population (point (c) in section “Energetics”), both mitochondria and peroxisomes contributed to the oxidation of the LCFA in GC B cells. In all, the findings indicate that GC B cells are programmed to support substantial rates of glucose consumption, anaplerotic energy generation, and FAO and suggest that the overall budget of energy sources may be similar for cells at each of the three stages (naive, activated, and GC B cells in the anti-NP repertoire). That said, direct assays of glycolysis or glucose oxidation, as in [70, 71] but in the presence and absence of other potential fuels, are needed to obtain a genuine quantitation of the energy and carbon budgets of these cells.
- Oxygen: molecular oxygen is a limiting factor in lymphoid follicles and even more markedly in the GC. Work conducted in parallel by three groups used intravital labeling that covalently modifies cells experiencing hypoxia (be it intracellular, extracellular, or both) to show hypoxia-enhanced signals in portions of most (but not all) GCs of immunized mice [78, 101, 225], with positive but weaker signals in follicles. A portion of GC B cells (in vivo and freshly sorted ex vivo) also exhibited stabilization of the α subunits of hypoxia-inducible transcription factors (HIF-1 and HIF-2) [78, 101]. Whether stabilization was due only to hypoxia is not clear since nonhypoxic HIF induction (including in B cells) has been well established. Experiments with B cells in vivo as well as in vitro provided evidence of a B cell-intrinsic mechanism in which persistent HIF stabilization reduced AID levels and altered Ab class switching mediated by mTORC1 [78], a step now thought to be mostly executed prior to GC entry or formation [226]. Of note, modeling these levels of hypoxia in vitro confirmed the expectation that oxidative metabolism would be maintained but underscored the potential for crosstalk among mechanisms, as hypoxia decreased amino acid uptake and mTORC1 activity [78]. HIF stabilization also dramatically impaired affinity maturation, notably that of IgG1, for which the all-affinity anti-NP response was unaffected [78]. Conversely, elimination of HIF-1α and HIF-2α expression yielded data suggesting that HIF in B cells can promote Ab responses [227]. While there are issues pertaining to the duration and level of HIF stabilization, the data strongly suggest that hypoxia influences B cell function among at least the GC subset that is hypoxic (~80% of those quantified in spleens). This model is supported by new work providing evidence that Cγ1-Cre- and *Cd21*-Cre-driven deletion of *Hif1a* decreased GC and anti-NP antibody responses [228]. Nevertheless, further studies and elucidation of the mechanisms that operate after HIF-1, HIF-2, or both are selectively inactivated within GC B cells are needed.

Other uncertainties remain in relation to the hypoxia in the majority of the GC. In principle, the reductive environment that engenders azole modification might be purely intracellular; e.g., oxygen consumption by mitochondria and peroxisomes (as noted in section “Energetics”) outpaces intracellular supply within B cells. However, pimonidazole staining and an mTORC1-dependent increase in HIF-1 in Tfh cells have been observed ([227, 229], and Huang B, Schwartzberg PS, personal communication). This result is consistent with the possibility that the GC on the whole may be relatively distant from capillaries [225] such that external oxygen delivered after unloading from hemoglobin is partially consumed by cells between the closest vessel and GC lymphocytes. Another uncertainty is to what extent hypoxia and HIF stabilization lead to HIF-dependent changes in gene expression programs (which themselves will be context-dependent, i.e., influenced by other signaling and transcriptional aspects of various GC B cells). The original observations included evidence that the expression of a gene set associated with the functional impact of hypoxia in certain cancer patients was enriched significantly in a polyclonal set of GC B cells relative to their IgD^+^ naive counterparts derived from a normal preimmune repertoire [78]. This enrichment—in a setting where, as noted, ~20% of the splenic GCs were pimonidazole-negative—has also been observed in several other RNA-seq datasets (summarized in [186]).

Other experiments have been performed using a system that facilitates recovery of increased numbers of hapten-binding B cells of the GC phenotype as well as generation of B cells activated by NP-Ficoll [87]. B cells with an Ig heavy chain that favors NP-binding BCRs and introgression of null alleles of Ig Vκ (because NP binding involves λ light chain pairing with the B1-8i knock-in transgene) were transferred in large numbers into a BCR transgenic mouse line that minimizes bystander inclusion in the GC. Gene set enrichment analyses with both a hypoxia signature derived from the breast cancer-like line MCF7 and another cancer-related gene set failed to achieve statistical significance when this transfer system was used [87]. A mini-meta-analysis tested how the adoptive transfer/restricted repertoire system compared informatically to prior gene expression data from independent laboratories using normal mouse lines. The salient result was how very different the B1-8i, Vκ–/– B cells were from those of normal mice [186]. The difference in findings may have arisen because the characteristics of B1-8i, Vκ–/– B cells in recipients with an allelically restrictive, nonreactive BCR created one circumstance in which the GCs that developed were programmed to conform to 20% of GCs in SRBC-immunized mice in which no hypoxia signal was detected [78]. Another potential explanation is that the difference in results arose due to intraclonal competition, as previously reported for the B1-8i system [230]. Apart from how BCR characteristics may influence the nature of GC B cells, an unanswered question about GC hypoxia, its variations, and the disparate informatics findings is how much gene expression or other characteristics are affected by bystander B cells and the relative balance among cells of varied fates in GC. Overall, take-away points are that (i) as with other aspects of GC reactions, there is variation [184, 197]; (ii) better and more GC B cell type-specific hypoxia and HIF regulatory signatures are needed; and (iii) additional work will be needed to establish the extent to which HIF stabilization in the full repertoire of normal B cells persists long enough to change GC outputs.

## The end of the affair—building and fueling the antibody factories

Apart from death, quiescence as memory B cells, or continuation as GC B cells, the alternative choice available to GC B cells is to become antibody-secreting plasmablasts and terminally differentiated plasma cells. Although lymphoblasts have to double their weight for each round of cytokinesis, a special challenge for plasma cells is that measurements of their secretion rate—while varied—indicate that they release as much as ¼–½ of their entire weight as secreted glycoprotein every day. Accordingly, plasma cells are programmed to meet this singular biosynthetic need, in contrast to rapidly dividing cells whose energy use and building blocks (carbon, nitrogen, oxygen) have to be distributed among a broader range of macromolecules (nucleic acids, lipids, protein). Excellent recent reviews cover the ER stress posed by this massive use of secretory protein pathways and the molecular mechanisms used to meet the challenge (e.g., [6, 7]).

### Signals stimulating the change

Initially, plasmablasts appear to move into the bloodstream from tissue sites [231–233]. High BCR affinity and T cells help push toward this outcome [58]. As discussed above, it is appealing to infer a direct connection to the capacity of ERK to promote the transition to a plasmablast/plasma cell fate via BACH2 [158, 160, 161, 211]. IL-4 and IL-21, acting in part via STAT3, also help drive plasma cell differentiation [234–236]. In addition, mechanisms sensing nutrient and energy status and the pathways connected with them are among the major regulators of the transition from B cells to plasma cells. Recent work indicates that B cells lacking both GSK3 isoforms (α and β) αρε are defective in their ability to contribute to a plasma cell population in an antigen-specific response (101, 192). As outlined in section “Signals and substrates for biosynthesis,” PI3K-activated mTORC2 regulates GSK3. However, earlier data showed an equally dramatic decrease in antigen-specific Ab level elicited by immunization of mice whose B cells were acutely depleted of Rictor to inactivate mTORC2 [133]. Thus, mTORC2 is unlikely to be a basis for the GSK3 findings, or vice versa, since mTORC2-AKT and GSK3 participate in reciprocal inhibition.

Downstream of these signaling pathways—and in principle subject to modulation by changes in the levels of metabolites in nuclei—gene regulatory networks specify the plasma cells and their specialized program that includes the need to adjust ER stress responses to a massive secretory load (reviewed in [59, 237]). The gene regulatory networks are established, maintained, and influenced by changes in DNA methylation and histone posttranslational marks that include acetylation and methylation ([238–242]; reviewed in [243]). Several salient points pertaining to cytosine methylation (mC) stand out. (i) The overall densities of mC decrease during the progression to plasma cell status [238–240]. (ii) As a cautionary note, even within different sources of plasma cells, the methylome can change substantially when the transcriptome does not [39]. (iii) Nonetheless, cell cycle-dependent conversion to hydroxymethylcytosine and full demethylation have been observed at regulatory elements for specific genes crucial in the plasma cell differentiation program, such as *Prdm1*, which encodes Blimp1 [239]. The detection of hydroxymethylation is of particular note because it implicates the actions of TET proteins such as TET2, and these αKG-dependent dioxygenases can be regulated by the accumulation of endogenous metabolites such as succinate, fumarate, and 2-hydroxyglutarate ([244, 245]; reviewed in [243, 246]). At present, the potential for such regulation to take place in a physiological setting, such as in the conversion of a GC B cell to a plasmablast or during plasmablast progression to a plasma cell, remains to be established, as does a mechanism targeting demethylases to specific regulatory elements.

### Nutrients, biosynthesis, and energetics

Most details regarding this broader topic, which has received less attention than its importance merits, have evolved little since excellent reviews have been published in the past few years [6, 7]. Accordingly, the summary here will be less expansive.

- Glucose: direct comparisons of plasmablast and plasma cells to other B lineage cells in spleen and marrow indicate that 2-NBDG uptake in vivo is greatly increased at these terminal stages (Brookens SK, Boothby MR, unpublished observations). Although actual glucose uptake may differ from what is measured with 2-NBDG, the increase is so dramatic as to imply that glucose use by plasma cells is far greater than that by B cells. Landmark work on SLPCs and LLPCs has indicated that they differ in their NBDG uptake [247, 248], although whether this is reflected by radiolabeled glucose is not clear. Stable isotope tracing with ^13^C- and ^14^C-glucose has provided evidence that much of this hexose ordinarily is diverted into providing substrates for glycosylation of secreted Abs [247]. That being the case, glycolytic sources of pyruvate appear to be relatively low in PCs, with a low fraction of energy likely to come from glucose oxidation. An arcane caveat relating to the overall patterns of glucose usage derives from evidence that when mannose is present at physiological concentrations, it can provide the main source of sugars for glycosylation [249]. An inference from this point is that the absence of mannose from the medium in tracing experiments could cause a greater fraction of glucose to be directed toward antibody glycosylation.
- Glutamine: as noted above (point (b) in section “Nutrient supply, uptake, and usage”), the plasmablasts elicited by Plasmodium infection in mice were highly glutamine-avid [219], indicating a high rate of uptake and suggesting that the large wave of such cells elicited by infection may have depleted this key amino acid locally. Such a mechanism would be consistent with the data and conclusions underscoring a key role for glutamine in B cell proliferation [91, 94].

### How long does the show go on (and why)?

Although the long duration of antibody circulation after administration of the best vaccines [250] has been appropriately emphasized, more granular analyses have shown that the plasma cells essential for maintaining circulating concentrations of antigen-specific Abs, LLPCs, are far from monotonic in longevity ([251–256]; reviewed in [257, 258]). Indeed, recent studies in the setting of SARS-CoV-2 infection have provided evidence of both Ag-dependent (anti-S vs. anti-N) and Ig class-dependent differences in persistence of Ab concentrations well after the plasmablast and SLPC phases, (e.g., [259]). Fundamental evidence has provided insights into different molecular programs evoked by different types of immunization such that the transcription factor ZBTB20 might be required or dispensable [260]. Moreover, the estimates of the masses of protein secreted by LLPCs differ substantially [247, 261, 262], and the localization of PCs or LLPCs that produce different classes of Abs varies [64, 65]. IgA-secreting cells in intestinal sites may be hypoxic, whereas meningeal IgA PCs [263] tend to stay “buffered” at a pO_2_ of ~7–10% (~40 mm Hg [264]) after passage through oxygen-saturated blood (75–100 mm Hg), and IgG secretors tend to reside in the bone marrow (reviewed in [66, 265]). Although important initial work on this challenging topic has started [266], the consequences of the differences in Ig class, nutrient supply, transporter profiles, and cell physiology represent an important area for further investigation. In the meantime, relatively little is known about either the molecular determinants of plasma cell longevity in vivo or their relationships to nutrients or the programming of intermediary metabolism. Plasma cell numbers decrease in the absence of Mcl-1 [267], whose expression can be modulated via GSK3 and involves a glucose-sensitive pathway [194, 195]. Similarly, the plasma cell lifespan is abbreviated by loss of canonical autophagy through elimination of Atg5 [214] or, for LLPCs, by reductions in the mitochondrial capacity to import pyruvate for its oxidation and generation of acetyl-CoA [247]. Conversely, lack of AMPK activity, probably due to increased mTORC1, has been reported to increase antibody production rates without impairing PC longevity or changing secretion efficiency [96]. An additional function of normal levels of mTORC1 may be to improve the programming of the ER and chaperones to facilitate PC function [138, 139]. These findings hint that in principle, the nutrient supply, flux of metabolites, and intrinsic programming of metabolism within an LLPC could influence its lifespan, along with niche competition and inflammation [257, 258, 268].

## Limitations and opportunities

The key limitation to highlight is the likelihood that we missed or inadequately represented some work that may be pertinent, even after input from peer referees. While efforts have been made to avoid such shortcomings, we cannot guarantee that we have succeeded. Moreover, a number of papers or possibilities have not been cited for reasons of space. A second limitation is that the work inevitably reflects the opinions or weighting of the authors, even after tempering by peer review. Some cognitive bias or inconsistency is likely present; for example, while RNA-seq, Seahorse assays, and even stable isotope metabolomics have major virtues, they can also provide distorted views of how cells are in fact working in situ.

The area is understudied, and vast amounts of basic information are needed that will likely inform analyses of sustained production of pathological autoantibodies secreted both by GC-derived plasma cells and those of extrafollicular origins (reviewed in [269–271]). These insights are likely needed for the identification of new therapeutic windows that will allow effective reductions in pathological autoantibodies without loss of the essential protection afforded by vaccines and infection-induced humoral memory. Another major frontier is the need for insight into nutrient and metabolic regulation of the functions of B cells, tertiary lymphoid structures, and antibody repertoires in cancers (reviewed in [4, 272–274]). Much of the mechanistic work is based on mouse systems, but analyses with humans and genetic variants, akin to those identifying how succinate dehydrogenase variants alter B cell physiology via a mitochondrial effect [275], are needed. For all of these issues, a pitfall of the current information and approaches lies in the fact that substrate supply, allostery, and product feedback inhibition regulate flux and steady-state concentrations of metabolites or biomolecules that are end-products of intermediary metabolism. In practice, this implies that techniques for imaging these molecules in situ, ultimately at single-cell spatial resolution, will be essential. Early application of such techniques has revealed an unexpected enrichment of peroxisome-dependent phospholipids in GCs [276], paralleling evidence of increased peroxisomal protein levels in GC B cells [87]. Along with developing fluorophore sensors to detect signaling in intact cells, advancing this technology to support determinations of intra- and extracellular nutrients and metabolites will enable analyses of variance versus uniformity in precursor-product relationships in vivo.
